# Farmed-raised fallow deer (*Dama dama* L.) carcass characteristics and meat nutritional value

**DOI:** 10.1007/s13197-020-04352-2

**Published:** 2020-03-18

**Authors:** Tomasz Żmijewski, Monika Modzelewska-Kapituła, Janusz Pomianowski, Agata Ziomek

**Affiliations:** Department of Meat Technology and Chemistry, Faculty of Food Sciences, University of Warmia and Mazury in Olsztyn, Plac Cieszyński 1, 10-719 Olsztyn, Poland

**Keywords:** Chemical composition, Fatty acids, Mineral compounds, Venison

## Abstract

This study analysed carcass features and the chemical composition of *semimembranosus* (SM) and *longissimus thoracis et lumborum* (LTL) muscles from farmed-raised fallow deer (*Dama dama* L.) bucks (n = 8). Mineral contents and fatty acid composition were also determined in the muscles and the fulfilment of the demand for minerals was calculated for adults. Dressing percentage was 55.2%, whereas the proportions of round, shoulder and loin were 38.4%, 15.5% and 14.4%, respectively. The highest content of meat tissue was found in round, followed by loin. There were no differences in moisture, fat, protein or ash contents between SM and LTL muscles, however differences in mineral contents and fatty acid composition were noted and affected the concentration of nutrients. Meat from farmed-raised fallow deer is a good source of iron and copper in the human diet and may be recommended as a part of a healthy diet.

## Introduction

The first modern fallow deer (FD, *Dama dama*) farm, dedicated to meat production, was established in 1969 in New Zeeland (Asher 1986). In Poland, commercial deer farms might have been established since 2001 according to the changes in the Act on the organization of breeding and reproduction of farm animals, which included FD and other deer species, which could be bred in captivity, to the farm animals group (Polish Law Gazette 2001 no. 129, position 1438). Currently, it is permitted to breed a red deer, sika deer and FD on farms (Polish Law Gazette 2007 no. 133, position 921). FD is the most popular deer species, and it is raised on 80% of wild animal farms (Kilar et al. 2015). In 2016, the population of FD accounted for 12,000 heads, whereas the total farmed deer population reached 18,100 heads (unpublished Federation of European Deer Farmers Associations data 2019). FD is also successfully farm-raised around the world and has gained interest due to the increasing demand of consumers for free-range meat products. This has been caused by the constantly increasing awareness of consumers regarding meat production on an industrial scale, especially its high impact on the environment and animal welfare. Moreover, raising deer on farms enables regular supplies of consistently high quality meat (Kudrnáčová et al. 2018).

There are numerous factors affecting slaughter value and the meat quality of FD, including life environment, slaughter age and diet. Since slaughter age significantly affects the FD carcass and meat quality, the animals are usually slaughtered between the 15th and 24th month, due to the highest body weight gains, the most effective feed conversion, low subcutaneous fat cover and the highest quality of meat (Janiszewski et al. 2014). The cold dressing proportion of FD carcasses, which indicates the economic value of carcasses, decreases with the age of animals. Żochowska-Kujawska et al. (2019) noted significantly higher dressing percentages for carcasses of 18-month-old FD than 42-month-old FD, and found no differences between 18 and 30 month-old bucks. In contrast, Volpelli et al. (2002) highlighted the economic benefits of the extension of FD breeding from 18 to 30 months, due to higher dressing proportions, higher amounts of first quality cuts and better carcass conformation. Moreover, the changes in body weight during the winter should be taken into consideration when choosing the appropriate slaughter age. Janiszewski et al. (2008) reported that the body weight of 18-month old FD before and after wintering did not differ, whereas a significant decrease was recorded after the winter in the body weight of animals older than 24 months. The above-mentioned facts justify the choice of 18-month-old FD as the material for the study. Moreover, the bucks were chosen due to the fact that an FD herd, does prevail with a minimum number of bucks, which are used for meat production (Janiszewski et al. 2014).

The significant role of the animal diet on the slaughter value of carcasses and meat quality was reported by Kudrnáčová et al. (2019), Volpelli et al. (2003) and Wiklund et al. (2005). In this study, farm-raised FD were pasture-fed, however, during autumn and winter periods their diet was supplemented with hay, silage and concentrate mixtures, which might have affected carcass and meat quality and produced results which were not described in previous papers.

Knowledge of the carcass composition and nutritional value of meat is crucial to promote it among nutritionists and consumers. Although FD is one of the most abundant deer species raised under farm conditions (Kudrnáčová et al. 2018), only a few studies have focused on the characteristics of main carcass cuts and nutritional value of FD meat. Due to variations in meat quality between wild and farm-raised FD and between the meat originating from buck and doe carcasses, the available data is incomplete. Daszkiewicz et al. (2015) reported significant differences in fatty acid composition between wild and farm-raised FD, with the wild animals producing meat with more desirable fatty acid composition and sensory properties, whereas Piaskowska et al. (2015) reported that muscles cut out from the carcasses of wild 18-month-old bucks had a higher content of dry matter, protein, fat and energy and were more beneficial from nutritional perspective fatty acid composition in intramuscular fat (higher C18:3 proportion in total fatty acids and higher ratio of polyunsaturated fatty acids to saturated fatty acids) than those from doe carcasses. Moreover, there is a shortage of information about the covering of demand for mineral compounds by the consumption of 100 g of FD meat and individual fatty acid intake, which is of great importance from a nutritional perspective. Therefore, the aim of the study was to determine the carcass composition of farmed 18-month-old FD and the nutritional value, including fatty acid and mineral concentrations, of two of the most valuable muscles: *m. semimembranosus* and *m. longissimus thoracis et lumborum.*

## Materials and methods

### Animals

The material for the study consisted of eight carcasses of fallow deer (*Dama dama* L.) bucks (approx. 18 months) raised from birth to slaughter in a farm located in north-eastern Poland (54°13′49″N 22°20′37″E). The area of the farm was approx. 300 ha. The stocking density of the animals was 10–15 deer/ha, whereas the structure of the flock’s sex was 1 buck per approx. 25 does. The animals were raised extensively; during summer they grazed on a natural pasture, which was composed of mostly grass with herbs. Moreover, they bit numerous bushes and trees found on the farm (willow, poplar, alder), constituting their additional fodder. In autumn, for 3 month prior to slaughter, special prepared stalls with a previously sown maize were available to animals. They ate both leaves and flasks. During winter, for 2 months prior to slaughter, animals were fed hay and silage ad libitum, and concentrate mixtures (oats with field pea) in an amount of approx. 0.5 kg/animal/day. The composition of the fodder fulfilled the recommendations: 50–70% dry matter, energy value from 2.3 to 2.6 MJ/kg, protein 13% dry matter, fibre from 18 to 22% (Janiszewski et al. 2014). Throughout the year, animals had free access to ordinary salt licks and those enriched with micronutrients, especially selenium.

Animals were harvested in January on the farm in accordance with Regulations of the Minister of Agriculture and Rural Development of 9 September (2004), with a shot with a rifle in the neck area, which enabled a rapid and proper exsanguination due to damage to the carotid arteries and zygomatic veins. Such a location of the gunshot wound also preserved the most valuable cuts of the carcass and did not reduce the quality of meat. After slaughtering, animals were weighed and morphometric measurements were made, including body length, height at the withers and chest circumference. The carcasses were eviscerated immediately after measurements (15–20 min from slaughter) and internal (kidney) fat was also removed. The skin with the head and lower limb sections were then removed. The carcasses were cooled at 4 °C ± 1 °C for 48 h and were then divided into cuts, which were then subjected to a detailed dissection.

At each stage of a carcass dissection, cuts, slaughter by-products and individual tissues were weighed. Based on the obtained results, the slaughter yield, percentage share of intestines and internal organs, skin, head, lower parts of limbs and kidney fat in respect to the weight of animals were calculated, as well as the share of individual cuts in the carcass and tissues in individual cuts and in the carcass. Additionally in two of the most valuable carcass cuts, *m. semimembranosus* (SM) and *m. longissimus thoracis et lumborum* (LTL), moisture, protein, fat, ash, total collagen and mineral (K, Na, Ca, P, Fe, Mn, Cu, Mg) contents and fatty acid composition were determined.

### Morphometric measurements

Directly after slaughter, the following measurements were made on intact carcasses using metric tape (± 1 cm): side length (from the occiput to the base of the tail), height at the withers (from the highest point above the shoulder blade to the end of the front leg), circumference of thorax (in the widest part of the thorax).

### Post-slaughter processing and dissection

**Fig. 1:**
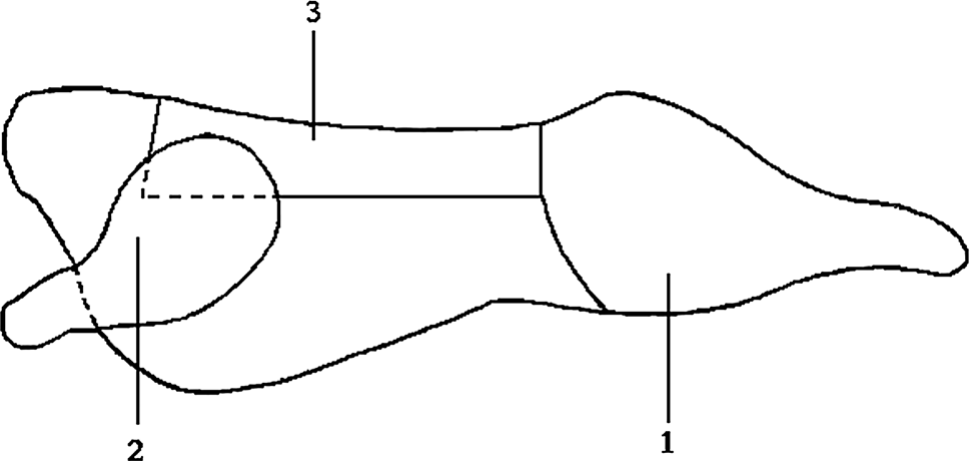
Cuts of fallow deer carcass: (1) round, (2) shoulder, (3) loin

As part of post-slaughter processing, evisceration and skinning were performed. Evisceration consisted of removing internal organs located in the abdominal and thoracic cavity of the animal. Skinning included removal of the skin with the head in the occipital joint and lower limb sections in the ankle and wrist joints. The carcasses were then jointed according to the method proposed by the Polish Industrial Standard (BN-84/9241-10), cut into the following cuts: (1) round—pelvic part of the half-carcass with the upper part of the hind leg, cut from the half-carcass from the front, between the penultimate and last lumbar vertebrae and further on the quadriceps muscle *epimysium*, from the top according to the central dividing line of the sacrum bone, from the bottom in the hock joint; (2) shoulder—the upper part of the forelimb from the wrist joint, cut from the thorax by a semi-circular incision according to the anatomical shape of the shoulder blade, running through the muscles connecting the forelimb with the thorax; the blade cartilage was left in the cut; (3) loin—the dorsal-lumbar part of the carcass cut off from the front perpendicularly to the spine between the 2nd and 3rd rib, from the back on the round cut-off line, between the penultimate and the last lumbar vertebrae, from the top on the half carcass cutting line, from the bottom by the straight, parallel cut to the spine, 4–5 cm from the lower edge of the *longissimus thoracis et lumborum* muscle; tenderloin was left in natural connection with the loin (Fig. 1). The parts of the carcass, which remained after cutting off the round, shoulder and loin, containing neck, ribs, flank and brisket (NRBF) were evaluated together.

Each cut was subjected to a detailed dissection into: lean meat (whole muscles, groups of muscles and lean trimmings with no visible fat particles, fat (subcutaneous and intermuscular), bones (all bones of a particular cut), connective tissue (tendons and thick fascia).

### Proximate chemical composition

Moisture content was determined according to PN-ISO 1442 (2000) by drying the samples at 105 °C to a constant weight; protein content according to AOAC (2006b, no. 992.15) using Kjeldahl’s method (N × 6.25); fat content according to AOAC (2006a, no. 991.36) using Soxhlet’s method with petroleum ether as a solvent; ash content according to PN-ISO 936 (2000) by ashing samples in quartz crucibles in a muffle furnace for 16 h (raising the temperature gradually from 150 to 600 °C); total collagen according to PN-ISO 3496 (2000) by determination of hydroxyproline content and coefficient 7.25. The energy value of meat was calculated using individual energy factors for protein (4.0 kcal, 16.78 kJ) and fat (9.0 kcal, 37.62 kJ).

### Mineral contents

#### Sample mineralization

The ashes were heat-dissociated in a mixture of nitric and perchloric acids (3:1) in an aluminium electric heating block with a temperature programmer (Velp Scientifica DK 20, Italy). The mineralization of samples was carried out for 4 h, gradually increasing the temperature from 120 to 200 °C. The colourless and clear mineralizate obtained was transferred to 50 cm^3^ graduated flasks and made up with deionized water up to the mark. Reagent samples were prepared parallel to the test samples.

#### Determination of Ca, Cu, Fe, Mg, Mn contents

The concentration of minerals in mineralizates was determined using the flame atomic absorption spectrometry method (flame:acetylene:air) with a Unicam 939 (Solar, UK) equipped with an Optimus data station, background correction (deuterium lamp) and cathode lamps suitable for each mineral. In Ca content determination, a 10% Cl_3_La · 7H_2_O (Merck, Germany) solution was added to all samples and standards to provide 1% of La^+3^ concentration in solutions, to eliminate the effect of phosphorus on the results.

#### Determination of Na and K contents

Contents of Na and K were determined by the emission technique (acetylene-air flame). Determinations were made using an atomic absorption spectrometer operating in an emission system (Py29 Unicam, UK).

#### Determination of P

Phosphorus in the analysed mineralizates was determined by the colorimetric method. Phosphates present in mineralizates were converted by molybdate (VI) in an acidic environment to phosphoromolybdate and then reduced with sodium (IV) sulphate and hydroquinone. As a result of the reduction, phosphoromolybdate was formed, whose concentration was determined calorimetrically. The absorbance measurement was performed using a spectrophotometer (Spectrophotometer VIS 600, Germany) at wavelength λ = 610 nm.

#### Fulfilment of the dietary recommendation for minerals

Fulfilment of the dietary recommendation for minerals covered by a consumption of 100 g of broiled FD meat was calculated based on the mineral content in raw meat. The percentages of the recommended daily allowance (RDA) of Mg, Ca, Fe, P and Cu and the Adequate Intake (AI) of K, Na and Mn were determined assuming a 40% loss of meat weight during heat treatment (Domaradzki et al. 2017). The following retention factors for minerals were used: Zn and Cu-1.0; Ca and Fe-0.95; and K, Na and Mg-0.85 (USDA 2007). Due to the lack of available literature data, losses during cooking were not taken into account for Mn and a retention factor of 1.0 was used (Domaradzki et al. 2017). Due to the lack of data for FD meat, coefficients for broiled beef cut were adapted (USDA 2007). Percentages of RDA and AI were calculated for adults (males and females, aged 19–50 years) based on the Institute of Medicine of the National Academy recommendations (IOM 2010).

### Fatty acid composition

To determine qualitative fatty acid composition in the muscles, lipids were cold extracted with the chloroform/methanol (2:1 v/v) method according to Folch et al. (1957). Fatty acids were trans-esterified with a chloroform:methanol:sulfuric acid (100:100:1) mixture using Peisker (1964) method. A chromatographic separation was performed using an Agilent Technologies 6890 N gas chromatograph (Agilent Technologies, Inc., Santa Clara, CA, USA) with a flame-ionization detector and a 30-m long, 0.32-mm internal diameter fused silica capillary column (matrix active group: poly(ethylene glycol) phase, Supelco, Bellefonte, PA, USA). The liquid phase was Supelcowax 10, and the film thickness was 0.25 μm. The separation was conducted under following conditions: carrier gas: helium; flow rate: 1 ml/min; detector temperature: 250 °C; injector temperature: 225 °C; column at temperature 180 °C. The detector signals were recorded using a Philips recorder with a 1 mV scale at a tape speed of 10 mm/min. The different acids were identified by comparing retention times with standards from Supelco (Bellefonte, PA, USA). The fatty acid content was presented as the relative percentage (% total fatty acids). The concentration of fatty acids in the meat (mg/100 g meat) was calculated based on a fat content and coefficient of 0.944 adopted from beef (Kunachowicz et al. 2005).

### Statistical analysis

The results obtained in all measurements were presented in the tables as mean values and standard error of mean (SEM). The data were processed by one-way ANOVA. To compare mean values, Duncan’s test at *p* < 0.05 was used (Statistica 12, StatSoft Inc., Tulsa, OK, USA).

## Results and discussion

### Carcass characteristics

Morphometric characteristics and the slaughter yields of FD carcasses are shown in Table 1. Due to a lack of information about the FD carcass dimensions, it is hard to compare the results obtained in the present study with literature. Carcass measurements might provide important information about its conformation and can be useful for meat producers. For the same slaughter weight, the longer the carcasses is, the worse conformation it shows. Moreover, shorter carcasses (with better conformation) are more desired in the slaughterhouse since this facilitates evisceration and subsequent cutting operations (Serrano et al. 2019).

**Table 1:** Morphometric characteristic and slaughter value of fallow deer carcass

Attribute	Mean value (SEM)	Min.	Max.
Side length (cm)	94 (3)	87	107
Height at the withers (cm)	83 (1)	76	88
Thorax circumference (cm)	94 (2)	88	97
Slaughter weight (kg)	48.77 (1.75)	42.06	59.14
Hot carcass weight (kg)	27.09 (1.39)	22.78	35.20
Cold carcass weight (kg)	26.91 (1.39)	22.72	35.07
Cooling losses (%)	0.63 (0.14)	0.26	1.65
Intestines and internal organs (kg)	15.46 (0.61)	12.80	18.40
Skin, the head, and lower parts of limbs (kg)	6.09 (0.22)	5.22	6.80
Kidney fat (kg)	0.13 (0.01)	0.06	0.18
Proportion to the slaughter weight			
Hot carcass weight (%)	55.55 (1.15)	50.07	59.52
Cold carcass weight (%)	55.20 (1.15)	49.81	59.30
Intestines and internal organs (%)	31.71 (1.10)	27.84	37.76
Skin, the head, and lower parts of limbs (%)	12.48 (0.48)	10.77	14.81
Kidney fat (%)	0.26 (0.03)	0.14	0.39
Weight of particular carcass cuts			
Round (kg)	10.34^a^ (0.49)	8.56	12.95
Loin (kg)	3.88^c^ (0.25)	3.17	5.38
Shoulder (kg)	4.17^c^ (0.18)	3.46	5.21
NRFB (kg)	8.52^b^ (0.49)	7.09	11.54
Proportion to the cold carcass weight			
Round (%)	38.42^a^ (0.33)	37.65	39.65
Loin (%)	14.42^d^ (0.20)	13.24	15.33
Shoulder (%)	15.50^c^ (0.37)	14.24	17.46
NRFB (%)	31.66^b^ (0.36)	29.65	32.89

*SEM* standard error of the mean, *NRFB* combined cuts of neck, ribes, flank, brisket

^a–d^mean values in columns with different letters differ significantly at *p* < 0.05

Slaughter weight (49 kg) and hot carcass weight (27 kg) were higher than reported by Volpelli et al. (2002) for an 18-month male FD (42 kg and 24 kg, respectively), but lower than a hot carcass weight reported by Stanisz et al. (2015) for a 32-month male FD (33 kg). However, the dressing percentage, in this study, was lower than reported by Stanisz et al. (2015), Volpelli et al. (2002) and Wiklund et al. (2005) (55.5% vs. 63.3%, 57.7% and 67.2%, respectively), which was caused by a higher weight of internal organs and gastrointestinal tract in this study. In contrast, Kudrnáčová et al. (2019) reported similar slaughter and carcass weights and dressing percentage (45 kg, 23 kg and 51%, respectively) for a 17-month-old farmed-raised FD from pasture feeding.

Weights of particular carcass cuts and their proportion in respect to the cold carcass weight are shown in Table 1. Carcass cuts (loin, shoulder, round and NRBF) differed in weights, except for loin and shoulder, which were similar, and had different proportion to the carcass weight (*p* < 0.05). The highest proportion in the carcass was round (38%) followed by NRBF (32%) and shoulder (16%), whereas the lowest was loin (14%) (*p* < 0.05). Carcass composition and quality are of great importance for meat producers, abattoirs and distributors, because they determine the economic value of slaughter animals, which results from the fact that different carcass cuts have different commercial values (Kudrnáčová et al. 2018). However, a comparison of the results regarding main carcass cuts proportion and their tissue composition with other reports is difficult due to variations in the way the carcasses were cut in different studies. However, Stanisz et al. (2015) reported that the proportions of loin and shoulder in 32-month male FDs were 17.7% and 16.7%, respectively, which was higher than in this study (14.4% and 15.5%, respectively).

The amounts and percentages of lean meat, fat, bones and tendons in the whole carcass and in particular carcass parts are shown in Table 2. The highest proportion in the carcass weight was meat (68%), followed by bones (20%), fat (7%) and connective tissue (4%), including tendons, which was expected. Meat content ranged from 63% in the NRBF to 74% in the round, and significant differences between cuts were noted (*p* < 0.05). The proportion of bones ranged from 18% to 26%. Loin, which is regarded the most valuable carcass cut, had lower meat, higher bones and connective tissue (*p* < 0.05) and similar fat percentages compared with round. In general, deer species have a high lean meat:bone ratio (5.5:1 in FD and 5:1 in red deer) and a higher proportion of lean meat (from 66 to 83%) than more conventionally raised ruminant species (Kudrnáčová et al. 2018). However, lower lean meat:bone ratio was noted (3.4:1) in this study although the results are in agreement with the findings of Kudrnáčová et al. (2019), who reported a similar meat:bone ratio in 17-month old FD (3.42:1).

**Table 2:** Content (mean values with SEM in parentheses) of lean meat, bones, fat and tendons (kg, %) in the whole carcass and carcass cuts from farm-raised male fallow deer

Cut	Lean	Bones	Fat	Tendons
Weight (kg)				
Round	7.65^a, w^ (0.40)	2.01^b, w^ (0.08)	0.46^c, x^ (0.03)	0.22^c, x^ (0.02)
Loin	2.72^a, y^ (0.17)	0.80^b, y^ (0.06)	0.13^c, y^ (0.01)	0.22^c, x^ (0.01)
Shoulder	2.70^a, y^ (0.13)	1.09^b, y^ (0.05)	0.24^c, y^ (0.02)	0.14^c, x^ (0.01)
NRFB	5.36^a, x^ (0.32)	1.57^b, x^ (0.08)	1.06^c, w^ (0.07)	0.50^d, w^ (0.04)
Carcass	18.43^a, v^ (1.00)	5.47^b, v^ (0.25)	1.89^c, v^ (0.11)	1.08^c, v^ (0.07)
Proportion (%)				
Round	73.92 (0.62)^a, v^	19.43 (0.41)^b, x^	4.42 (0.26)^c, y^	2.12 (0.19)^d, x^
Loin	70.07 (0.27)^a, w^	20.64 (0.30)^b, w^	3.43 (0.29)^d, y^	5.70 (0.16)^c, v^
Shoulder	64.65 (0.56)^a, y^	26.00 (0.12)^b, v^	5.78 (0.47)^c, x^	3.44 (0.21)^d, w^
NRFB	62.92 (0.70)^a, z^	18.44 (0.43)^b, y^	12.48 (0.51)^c, v^	5.88 (0.32)^d, v^
Carcass	68.45 (0.46)^a, x^	20.31 (0.28)^b, wx^	7.04 (0.27)^c, w^	4.03 (0.10)^d, w^

*SEM* standard error of the mean, *NRFB* combined cuts of neck, ribes, flank, brisket

^a–d^mean values in rows with different letters differ significantly (*p* < 0.05)

^v–z^mean values in columns (within each way of results presentation) with different letters differ significantly (*p* < 0.05)

### Proximate chemical composition

SM and LTL muscles did not differ in dry matter, protein, fat, ash or collagen content (*p* > 0.05, Table 3). Volpelli et al. (2003) reported also similar contents of collagen, fat and ash in LTL and SM muscles of 18-month FDs. The results of this study resemble those reported by Daszkiewicz et al. (2015) and Bureš et al. (2015) for dry matter, protein and ash contents in LTL muscle. However, fat and collagen contents determined in the present study in LTL muscle (1.55% and 1.12%, respectively) were higher than reported by Daszkiewicz et al. (2015) in meat obtained from wild and farm-raised FD (fat content 0.50% and 0.24%, respectively) and Bureš et al. (2015) (0.72% and 0.32% for fat and total collagen, respectively) in LTL muscle from fallow bucks at a similar age to those used in the present study. The chemical composition of meat depends on animal’s age and diet (Volpelli et al. 2003). The differences between the present study and Bureš et al. (2015) might thus be caused by a different composition of animals diet—although all FD grazed on natural pastures, they also received a concentrate, which was different in different studies. Generally, pasture is a basic source of feed for farmed deer species, but a simple grain-based diet supplementation is a common practice to improve an animal performance, during winter or a season with harsh conditions (dry or wet and muddy season). Animals supplemented with grains have higher slaughter yields and proportion of individual carcass parts and more intramuscular fat (IMF) and carcass fat than pasture-fed animals (Kudrnácová et al. 2019). Volpelli et al. (2003) reported a higher fat content in LTL and SM muscles and a higher protein content in SM muscle obtained from FD fed with concentrate compared with pasture fed animals.

**Table 3:** Chemical composition and energy value (mean values with SEM in parentheses) of *semimembranosus* (SM) and *longissimus thoracis et lumborum* (LTL) muscles from farm-raised male fallow deer

Constituent	SM	LTL
Dry matter (%)	25.21^a^ (0.25)	25.96^a^ (0.62)
Total protein (%)	23.33^a^ (0.31)	23.21^a^ (0.51)
Collagen (%)	1.16^a^ (0.10)	1.12^a^ (0.10)
Fat (%)	0.79^a^ (0.15)	1.55^a^ (0.35)
Ash (%)	1.03^a^ (0.04)	1.10^a^ (0.04)
Water/protein ratio (W/P)	3.21^a^ (0.05)	3.20^a^ (0.10)
Energy value (kJ)	421.3^a^ (5.8)	447.9^a^ (16.6)

^a^mean values in rows with common superscript do not differ (*p* > 0.05)

### Mineral contents

SM and LTL muscles differed in the concentration of Na, which was higher in LTL, and Mg content and higher in SM, whereas there were no differences in K, P, Fe, Ca, Cu and Mn contents between the muscles (Table 4). There were slight differences in the percentages of the fulfilment of the dietary recommendations for mineral intakes between SM and LTL muscles (Table 4), which correspond to the concentration of minerals in the muscle tissue. The FD meat was a very good source of Fe and the consumption of 100 g of cooked meat satisfies 63–65% and 28–29% of RDA for males and females, respectively. The values of Fe content in FD meat (3.2–3.3 mg/100 g, Table 4) are relatively high when compared with other kinds of red meat. Williams (2007), also reported that the concentration of Fe in beef, veal and lamb ranges from 1.1 to 2.0 mg/100 g, whereas in mutton it reaches 3.3 mg/100 g. In addition to the high concentration of Fe in meat, the absorption of the mineral from meat is higher than from a plant food (Williams 2007).

**Table 4:** Mineral compounds content (mean values with SEM in parentheses) in *semimembranosus* (SM) and *longissimus thoracis et lumborum* (LTL) muscles from farm-raised male fallow deer and the fulfilment of the requirements of adults for mineral compounds satisfied by the consumption of 100 g of cooked meat

Mineral compound	Content of minerals (mg/100 g of wet tissue)		Gender, age group	Standard (mg/d)	% of the standard	
	SM	LTL			SM	LTL
K	386.76^a^ (4.39)	376.76^a^ (3.46)	♂♀ 19–50 year	4700	11.7	11.4
P	235.36^a^ (4.25)	231.36^a^ (3.90)	♂♀ 19–50 year	700	50.4	49.6
Na	49.50^b^ (1.13)	59.56^a^ (5.57)	♂♀ 19–50 year	1500	4.7	5.6
Mg	23.57^a^ (4.50)	21.64^b^ (0.41)	♂ 19–30 year	400	8.3	7.7
			♂ 31–50 year	420	8.0	7.3
			♀ 19–30 year	310	10.8	9.9
			♀ 31–50 year	320	10.4	9.6
Fe	3.18^a^ (0.09)	3.29^a^ (0.14)	♂ 19–50 year	8	62.9	65.0
			♀ 19–50 year	18	28.0	28.9
Ca	1.88^a^ (0.11)	2.27^a^ (0.17)	♂♀ 19–50 year	1000	0.3	0.4
Cu	0.17^a^ (0.02)	0.17^a^ (0.002)	♂♀ 19–50 year	0.9	31.9	32.2
Mn	0.02^a^ (0.002)	0.02^a^ (0.002)	♂19–50 year	2.3	1.3	1.0
			♀19–50 year	1.8	1.7	1.3

The  % of the standard met by 100 g of cooked meat was calculated on the basis of the mineral content in raw meat, assuming 40% cooking loss from the raw meat and the following retention factors: Ca, Cu-1, Fe-0.95, P-0.9, Mg, Na, K-0.85 (USDA 2007) and Mn-1; adequate intake (AI) for K, Na, Mn; recommended dietary allowance (RDA) for P, Mg, Ca, Fe, Cu. Source for nutrient requirements IOM (2010)

♂, requirements for males; ♀, requirements for females

^a,b^mean values in rows with different superscripts differ significantly at *p* ≤ 0.05

FD meat was also a rich source of P and Cu and the consumption of 100 g of cooked meat satisfies 50% and 32% of the RDA for adults, respectively, and is a relatively good source of Mg and K (up to 12% of RDA and AI, respectively). In the case of Mn, Na and Ca, low values were obtained ranging from 1.0 to 1.7% AI for Mn, from 4.7 to 5.6% of AI for Na and from 0.3 to 0.4% of RDA for Ca. The obtained results were not surprising, because meat is recognized as a marginal source of Ca and Mn in the human diet (Cabrera et al. 2010). However, low values of AI of Na, indicate that meat cooked without the use of salt FD meat will not contribute to excessive consumption of Na and heath disorders, such as increased blood pressure.

### Fatty acid (FA) composition

A total of 12 FAs were determined in SM and LTL muscles (Table 5). The predominant fatty acids were palmitic acid (C16:0), linoleic acid (C18:2, n-6), stearic acid (C18:0) and oleic acid (C18:1), which is similar to the findings of Bureš et al. (2015). The percentages of polyunsaturated fatty acids such as C18:2, n-6, C18:3 n-3 and C20:4 n-6 in this study were higher than reported by Daszkiewicz et al. (2015) for farmed FD and Piaskowska et al. (2015) for wild male FD.

**Table 5:** Fatty acid composition (% of total fatty acids and mg/100 g, mean values with SEM in parentheses) of intramuscular fat of *semimembranosus* (SM) and *longissimus thoracis et lumborum* (LTL) muscles and kidney fat from farm-raised male fallow deer

Fatty acid	Profile (% fatty acids)			Concentration (mg/100 g)	
	SM	LTL	Kidney fat	SM	LTL
C 14:0	1.26^b^ (0.12)	1.96^b^ (0.16)	4.75^a^ (0.41)	9.42^y^ (0.88)	28.68^x^ (2.37)
C 14:1	0.45^a^ (0.01)	0.45^a^ (0.14)	0.83^a^ (0.05)	3.38^x^ (0.02)	6.63^x^ (2.10)
C 15:0	0.89^b^ (0.02)	1.06^b^ (0.01)	2.51^a^ (0.20)	6.64^y^ (0.11)	15.56^x^ (0.21)
C 16:0	20.28^b^ (0.21)	22.00^b^ (1.26)	28.39^a^ (1.51)	151.22^y^ (1.56)	321.86^x^ (18.40)
C 16:1	2.85^b^ (0.41)	3.96^a^ (0.30)	1.78^c^ (0.02)	21.23^y^ (3.08)	57.94^x^ (4.33)
C 17:1	0.70^a^ (0.13)	0.76^a^ (0.10)	0.31^b^ (0.06)	5.25^y^ (0.98)	11.12^x^ (1.52)
C 18:0	18.81^b^ (0.17)	19.17^b^ (0.32)	41.67^a^ (1.53)	140.30^y^ (1.27)	280.54^x^ (4.60)
C 18:1 cis 9	14.34^b^ (0.36)	16.67^a^ (0.45)	15.90^ab^ (0.56)	106.94^y^ (2.65)	243.92^x^ (6.63)
C 18:2 n-6	22.95^a^ (0.54)	18.54^b^ (1.23)	2.19^c^ (0.19)	171.15^y^ (4.03)	271.23^x^ (17.96)
C 18:3 n-3	4.83^a^ (0.06)	4.72^a^ (0.26)	1.13^b^ (0.08)	36.02^y^ (0.48)	69.06^x^ (3.81)
C 20:0	ND	ND	0.54 (0.02)	ND	ND
C 20:4 n-6	10.16^a^ (0.40)	8.48^a^ (0.81)	ND	75.79^y^ (2.97)	124.03^x^ (11.92)
C 20:5 n-3	2.48^a^ (0.40)	2.24^a^ (0.25)	ND	18.49^y^ (2.95)	32.78^x^ (3.66)
Σ SFA	41.24^b^ (0.51)	44.19^b^ (1.74)	77.86^a^ (0.58)	307.58^y^ (3.83)	646.64^x^ (25.40)
Σ MUFA	18.34^b^ (0.91)	21.84^a^ (0.23)	18.82^b^ (0.47)	136.80^y^ (6.68)	319.61^x^ (3.30)
Σ PUFA	40.42^a^ (1.40)	33.97^b^ (1.91)	3.32^c^ (0.27)	301.46^y^ (10.42)	497.10^x^ (28.02)
n-6/n-3	4.55^a^ (0.16)	3.90^a^ (0.37)	1.93^b^ (0.07)	–	–

*SEM* standard error of mean, *SFA* saturated fatty acids, *MUFA* monounsaturated fatty acids, *PUFA* polyunsaturated fatty acids, *ND* not detected

^a–c^ and ^x–y^mean values in rows with different superscripts differ significantly at *p* ≤ 0.05

LTL muscle showed a higher proportion of palmitoleic (C16:1), oleic (C18:1) and monounsaturated fatty acids (MUFA), whereas a lower proportion of linoleic (C18:2 n-6) and the sum of PUFA than SM muscle. In the kidney fat, a total of 11 fatty acids were determined. The fat contained arachidic acid (C20:0), which was not detected in intramuscular LTL and SM, whereas arachidonic (C20:4 n-6) and eicosapentaenoic (C20:5 n-3) acids, which were detected in the IMF, were not detected in the kidney fat. Predominant fatty acids in the kidney fat were stearic (C18:0) and palmitic acids (C16:0). The kidney fat contained a higher proportion of saturated fatty acids (SFA), including myristic (C14:0), pentadecylic (C15:0), palmitic (C16:0), stearic (C18:0), and a lower share of PUFA, including linoleic (C18:2 n-6) and linolenic (C18:3 n-3) as well as MUFAs such as palmitoleic (C16:1) and margaroleic (C17:1) acids than IMF extracted from SM and LTL muscles. Differences in the FA composition of IMF and internal adipose tissue depots might be explained by the accumulation of long chain PUFAs in muscle phospholipid (Wood et al. 2008). Linoleic acid (18:2 n-6) is deposited in both adipose and muscle tissues, however in muscle tissue it is accumulated in phospholipids at a high level where it and its long chain products, e.g. arachidonic (20:4 *n*-6) and eicosapentaenoic (C20:5 n-3) acids, compete for insertion into phospholipid molecules (Wood et al. 2008). This explains the lack of C20:4 n-6 and C20:5 n-3 in kidney fat in this study.

Consumers are encouraged to choose a lean meat with a high proportion of PUFA due to their potential to reduce the low-density lipoprotein (LDL) cholesterol level and the risk of cardiovascular disease (EFSA 2010). The FA profile of LTL muscle studied in this study was more beneficial than reported by Bureš et al. (2015) due to a higher proportion of PUFA and lower n-6/n-3 ratio. The differences might be caused by a different concentrate composition and age of animals. On the other hand, Bureš et al. (2015) noted that the fatty acid composition of FD was more beneficial from a nutritional perspective than cattle (Aberdeen Angus and Holstein) because of a higher MUFA and PUFA proportion, including n-6 and n-3, and lower SFA, which may be useful in promoting this kind of meat among consumers. Moreover, as pointed out by Valencak et al. (2015), cooking does not substantially alter the favourable fatty acid composition of venison (red and roe deer). There were no significant differences (*p* > 0.05) in the n-6/n-3 ratio between SM and LTL muscles, whereas kidney fat had a significantly (*p* < 0.05) lower n-6/n-3 ratio than IMF. The ratio n-6/n-3 was used as an indicator of the nutritional value of fats and the value of 4.0 was set as the maximal (Valencak et al. 2015). However, more recently, WHO/FAO (2008) and EFSA (European Food Safety Authority) (2010) panels declined to set specific values for the n-3/n-6 ratio.

Differences in the fatty acid composition and fat content between SM and LTL muscles affected the concentration of fatty acids (mg/100 g) in the muscle tissue (Table 5). SM and LTL muscles differed in all determined fatty acid, except for C14:1, and higher content were found in LTL muscle. The concentration of PUFA in 100 g portion of LTL was 65% higher than in SM, whereas PUFA n-3, including C18:3 n-3 and C20:5 n-3, were 87% higher. On the other hand, the concentration of SFA was also higher in LTL muscle (110% of the concentration in SM muscle). General recommendations for fatty acid consumption indicates that the total intake of SFA should not exceed 10% energy, whereas PUFA should account for 6–11% energy. The minimum intake levels of linoleic (C18:2, n-6, LA) and alpha-linolenic (C18:3 n-3, ALA) acids, which are indispensable since they are not synthesized in the human body to prevent deficiency symptoms, are estimated at 2.5% of energy for LA plus 0.5% energy for ALA (WHO/FAO 2008). In view of these recommendations, and the results resented in Table 5, FD meat can be considered a low fat meal with a beneficial fatty acid composition.

## Conclusion

The carcasses of farmed-raised FD showed a high proportion of lean meat, which is vitally important for both meat manufacturers and consumers. Due to high variations in the quality of FD carcass and meat, resulting from different animal ages and feeding regimes, the results of this study broaden knowledge of this kind of animal production. The high content of mineral compounds (especially Fe and CU) and beneficial fatty acid composition make FD meat a valuable component of the human diet.
